# A Pancreaticobronchial Fistula Associated With Previous Trauma and Pancreas Pseudocysts: A Case Report

**DOI:** 10.4021/gr560w

**Published:** 2013-09-09

**Authors:** Sari Venesmaa, Petri Juvonen, Hannu-Pekka Kettunen

**Affiliations:** a Departments of Gastroenterological Surgery, Kuopio University Hospital, Kuopio, PO. BOX. 100, FI-70029 KYS, Finland; b Clinical Radiology, Kuopio University Hospital, Kuopio, PO. BOX. 100, FI-70029 KYS, Finland

**Keywords:** Pancreaticobronchial fistula, Abdominal trauma, Pancreatic pseudocyst

## Abstract

We describe a rare case of a pancreaticobronchial fistula caused by pancreatic pseudocysts due to previous trauma. A 54-year-old man with a history of traumatic hemothorax was referred to central hospital for investigations due to cough, dyspnea, vertigo and fever. An ultrasound scan and abdominal computed tomography scan showed huge pancreatic pseudocysts around the pancreas extending to the right side of the mediastinum with gas. The etiology for the pseudocysts was unconfirmed. First, the patient recovered with antibiotics and external pseudocyst drainage. After five months the patient started to suffer from respiratory symptoms again, such as coughing with sputum, dyspnea and mild fever. The computer tomography scan confirmed the pancreaticobronchial fistula as a diagnosis and the patient was referred to the university hospital for further treatment.

## Introduction

Thoracic manifestations of pancreatic disease are caused by acute disruption of the pancreatic duct posteriorly, resulting in the flow of pancreatic secretions into the retroperitoneum. The fluid may track up either the aortic hiatus or the esophageal hiatus, forming a pancreaticopleural fistula (PPF) or mediastinal pseudocyst [1]. Adhesion of the pleura of the lung to the diaphragm predisposes patients to the formation of a pancreaticobronchial fistula. However, rupture into the bronchial tree resulting in a pancreatico-bronchial fistula is a rare complication, and both conservative, endoscopic and surgical management have been advocated [2]. The cause of a pancreaticothoracic fistula has been attributed to chronic pancreatitis. Trauma leading to disruption of the pancreatic duct can also cause an internal pancreatic fistula, but this is the rarest etiological factor [3]. We report a case of a patient with pancreaticobronchial fistula probably caused by old trauma with hemothorax combined with pseudocyst formation.

## Case Report

**Figure 1:**
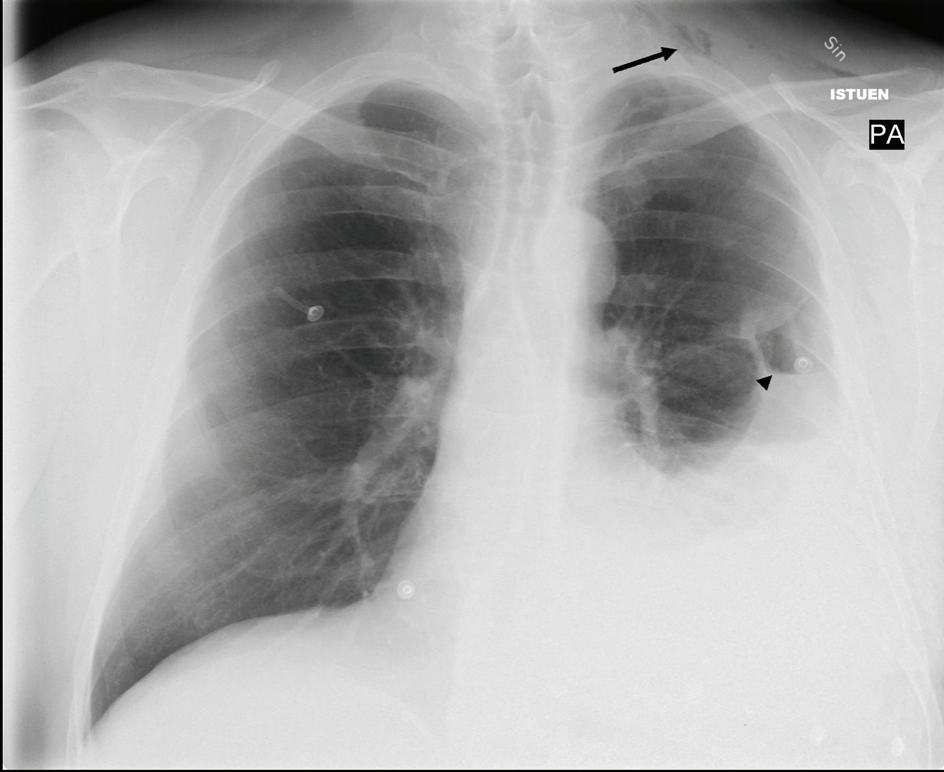
Chest X-ray showing left-sided pleural effusion with a small apical pneumothorax indicative of a hemo-pneumothorax. Note subcutaneous air in left jugular region (arrow) and a small air-fluid level (arrowhead).

A 54-year-old man was referred to the central hospital with a left-sided hemothorax. Two days earlier he had fallen and hurt his left flank, chest and back. His medical history included hypertonia and asthma. On admission, he had pain and tenderness on the left side of his abdomen and left flank. On physical examination, we observed a blood pressure of 100/60 mmHg, pulse 110/min (regular). Laboratory tests: hemoglobin (Hb) 114 - 101 g/L, hematocrit (Hct) 0.34 - 0.30. C-reactive protein (CRP) 81 mg/L. Chest radiography confirmed a left-sided hemothorax (Fig. 1) but his abdominal ultrasound scan was normal. The pancreas was not visualized by ultrasound. A left-sided chest drain was inserted and 2,400 mL of blood was extracted from the pleurae. A blood transfusion of 800 mL was given intravenously to stabilise the patient’s decline in hemaglobin. After four days in hospital the patient was asymptomatic and discharged.

A year and half later, the patient was again admitted to the central hospital with fever, cough and dyspnea that had started two weeks earlier. The patient would normally walk many kilometers without any symptoms. Before admission, he was unable to walk only one hundred meters because of his shortness of breath. He described the feeling of choking during recent nights. Over the previous month, his appetite had diminished. There was alcohol abuse in his recent history but no prior evidence of pancreatitis. On physical examination, the patient’s blood pressure was 141/102 mmHg, pulse 90/min (regular), T 37.6 °C. Laboratory tests: Hb 92 g/L, Hct 0.28, CRP 215 mg/L, white blood cell count (WBC) 10.3 × 10^9^/L, thrombocytes (B-Tromb) 663 E9/L, Erythrocyte Sedimentation Rate (ESR) 14 mm/h, plasma amylase (P-Amyl) 219 U/L. Alanine transaminase and alkaline phosphatase were only lightly elevated. Serum bilirubin, creatinine and urea measurements were all within normal ranges. Respiratory and heart diseases were excluded by chest radiography and electrocardiography. On admission, there was no tenderness or pain in the abdomen. The reason for this infection and fever was unknown for two days before abdominal ultrasound was performed. On ultrasound, fluid collections were seen in the upper abdomen and computer tomography (CT) was scheduled.

**Figure 2:**
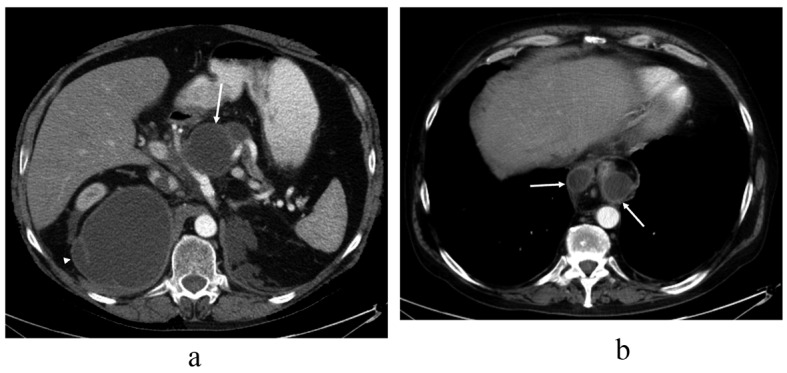
Contrast-enhanced abdominal CT image taken over 1.5 years after the thoracic injury, revealing (a) communicating fluid collections above the pancreas (arrow) and in the right subphrenic space (arrowhead) with wall enhancement. There is also a small separate fluid collection in the left subphrenic space indicative for old hematoma. The pancreas shows atrophy but there are no parenchymal calcifications or peripancreatic edema, so there is no evidence of acute or chronic pancreatitis. In the upper level (b) the pseudocysts (arrows) rise up through the hiatus opening to the mediastinum surrounding the esophagus. At this time, the lung parenchyma was normal. No pleural effusion is seen.

By abdominal CT, the fluid collections were found to be pseudocysts around the pancreas that ascended towards the mediastinum through the hiatus next to the esophagus (Fig. 2). The pseudocysts originated from the pancreatic body and were in contact with a normal sized pancreatic duct in the pancreatic tail. Another separate fluid collection was seen under the left diaphragm. The pancreas was atrophic but there were was no evidence of acute or chronic pancreatitis seen on the CT scan.

The patient was first treated by intravenous cefuroxime for five days and then by oral levofloxacin for seven days. The patient became apyrexial and his CRP decreased to 21 mg/L. The patient went home on day 6 post admission. Only ten days later the patient was admitted back to hospital with fever and his CRP was again elevated at 225 mg/L. On ultrasound, the pancreatic pseudocysts were unchanged and finally an external drain was inserted into the largest fluid collection by a radiologist. The fluid was a free-flowing liquid and brown in color. There were no bacteria grown by the fluid cultures and the CEA (carcino embryogenic antigen) was normal. The fluid amylase content was extremely high 32,920 U/L, which is strongly indicative of a pancreatic fistula. Three weeks after the external drainage of pseudocysts, the patient was asymptomatic and on ultrasound the right sided huge pseudocyst was disappeared. The patient was discharged home.

The etiology of these pancreatic pseudocysts remained open and MRCP (magnetic resonance cholangiopancreatography) was scheduled.

**Figure 3:**
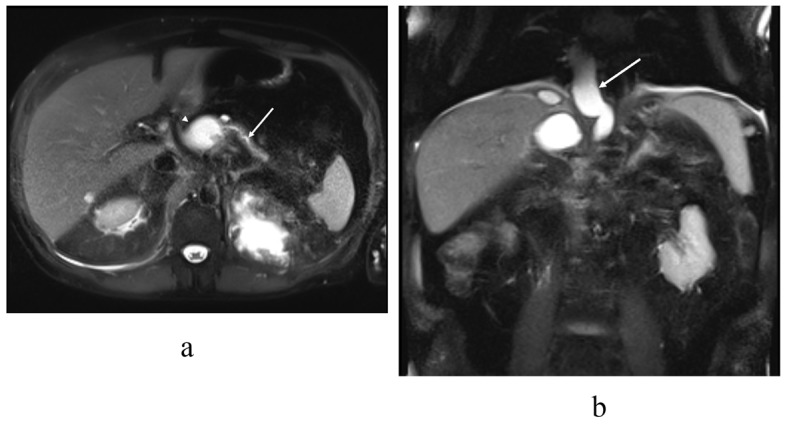
T2 (haste) MRI in the (a) transaxial and (b) coronal plane demonstrates the distal pancreatic duct (arrow) communicating with the pancreatic body pseudocyst (arrowhead). In the coronal plane (b) a pseudocyst (arrow) extends through the hiatus opening to the mediastinum, similar to the previous CT scan.

One month later, MRCP showed that the fluid collection was around the pancreas and the pancreatic duct was interrupted within the body of the pancreas (Fig. 3). The fluid collection protruded to the mediastinum but the lungs and pleurae were intact. The remaining fluid collection under the diaphragm was separated and deemed to be a chronic hematoma.

**Figure 4:**
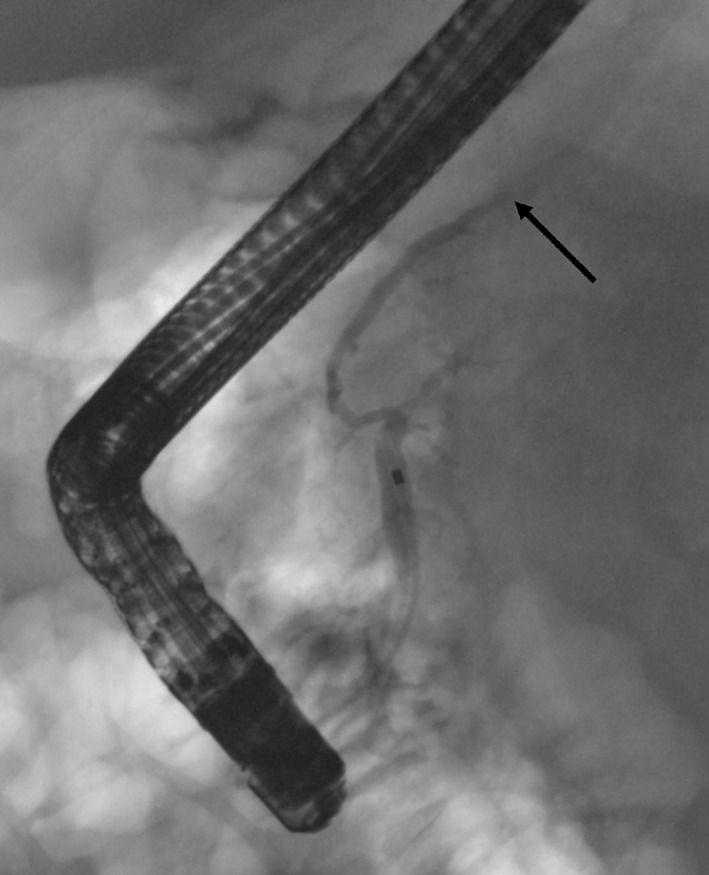
In the first ERCP the cannula is in the pancreatic duct. The duct appears normal at the head and the body but within the body-tail junction the duct is interrupted (arrow) and no contrast is seen in the pancreatic tail duct. No contrast leakage is seen at this time.

The patient was admitted to our hospital for endoscopic retrograde cholangiopancreatography (ERCP). The duodenoscopy (Olympus Evis Extra TJF-160VR) was transmitted to the duodenum and the papilla major was cannulated by passage of a cannula (4.5 Fr), and then ductography was performed. By radiography, the duct in pancreatic head was seen and deemed normal but the duct in body of pancreas was interrupted and no contrast was seen in the duct of the pancreatic tail. The pseudocysts around the pancreas were not filled with contrast either (Fig. 4) and so no stent was inserted into pancreatic duct. Laboratory tests: CRP < 5 mg/L, Hb 124 g/L, P-amyl 219 U/L. The patient was in good health and discharged home. A CT scan was scheduled in the central hospital for four months later.

**Figure 5:**
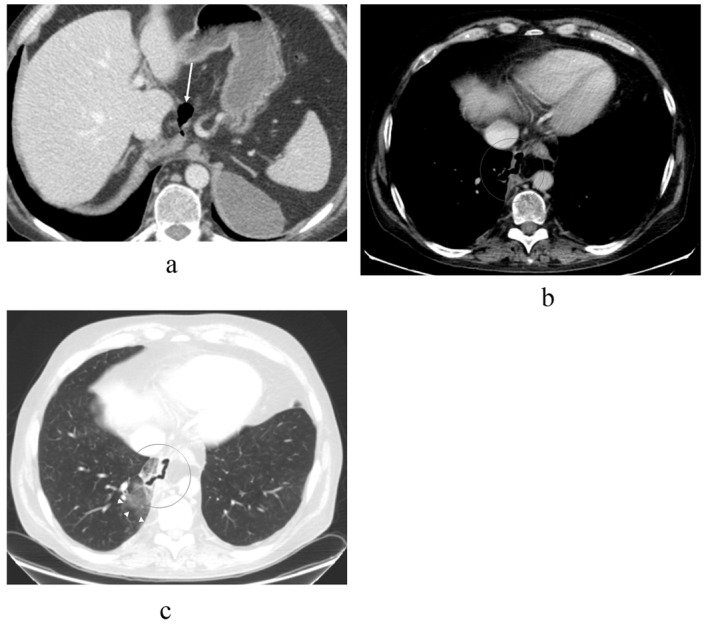
CT scan images taken 4 months after the primary ERCP shows that the pseudocysts have retreated (a) compared to previous CT and MRCP scans (Fig. 2, 3) but there is an air-filled passage above the pancreas (arrow), which protrudes in to the mediastinum. The separate left subphrenic hematoma is still visible. In the upper level with (b) mediastinal and (c) lung window settings the air-filled channel penetrates through the right mediastinal pleura in to the lung parenchyma where there is some ground-glass opacity (arrowheads) indicative of parenchymal irritation. No pleural effusion is seen.

During the next four months the patient had a bad cough, bitter sputum and mild fever. These symptoms were worst at night. He was treated by many antibiotics without a good response. Finally, by CT there was an explanation for these symptoms. A fistula containing gas tracked from the body of the pancreas and protruded into the mediastinum to contact the pulmonary parenchyma. Around the lower lobe of the right lung there were profuse inflammatory changes (Fig. 5). The diagnosis was a rare pancreaticobronchial fistula. The patient was admitted to our hospital for a new ERCP.

**Figure 6:**
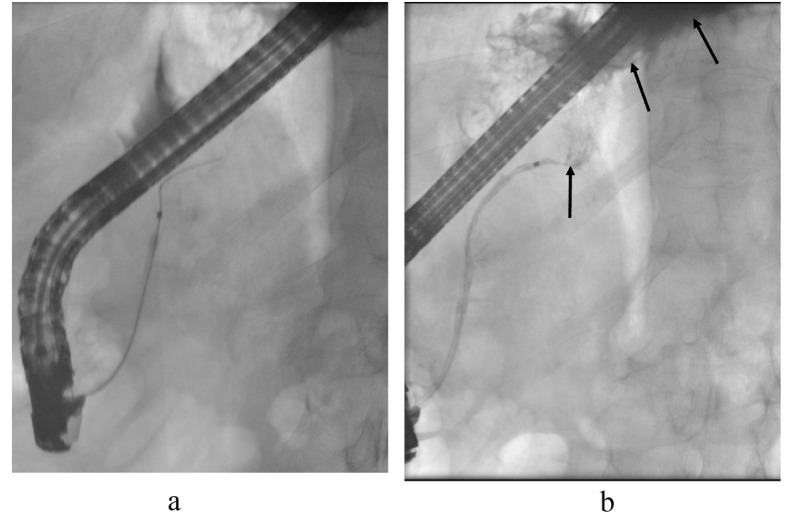
ERCP control images. The cannula is in the pancreatic duct. (a) After contrast injection, contrast leakage is seen above the pancreatic body (arrows) filling the mediastinal pseudocyst (b). The pancreatic duct was stented.

ERCP confirmed a disrupted pancreatic duct communicating with the mediastinal pseudocysts (Fig. 6). The pancreatic duct was stented with a 7 cm 5 Ch stent. During ERCP, the patient was coughing when contrast was injected to the pancreatic duct and mediastinal pseudocyst.

After one month, a control CT was taken and the inflammatory changes in the right lung had resolved. The stent was in place in the pancreatic duct and fluid collections were still seen around the pancreas. The patient was feeling better and no longer had a productive cough. Two months on, the patient still suffered from two infections with abdominal pain and pyrexia, which were treated in the central hospital with antibiotics. A CT scan revealed edema around the fluid collections, suggesting pseudocyst infection. In the laboratory tests, the CRP was 273 mg/L but decreased rapidly after starting antibiotics. The plasma amylase was normal. Because of these acute infections, the patient was in the central hospital for six days in total. After antibiotic treatment he was symptom free and was discharged.

**Figure 7:**
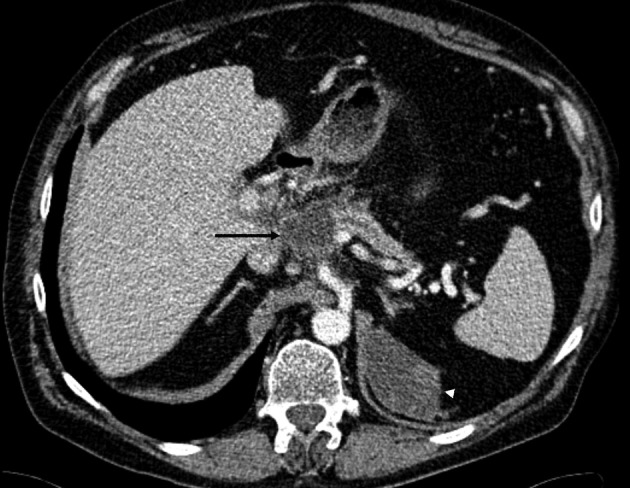
A contrast-enhanced CT scan image shows a small 4.3 cm residual pseudocyst (arrow) above the pancreatic body adjacent to the normal caliber pancreatic duct. There is peripancreatic edema indicative for acute pancreatitis. The left subphrenic chronic hematoma is slowly shrinking (arrowhead).

**Figure 8:**
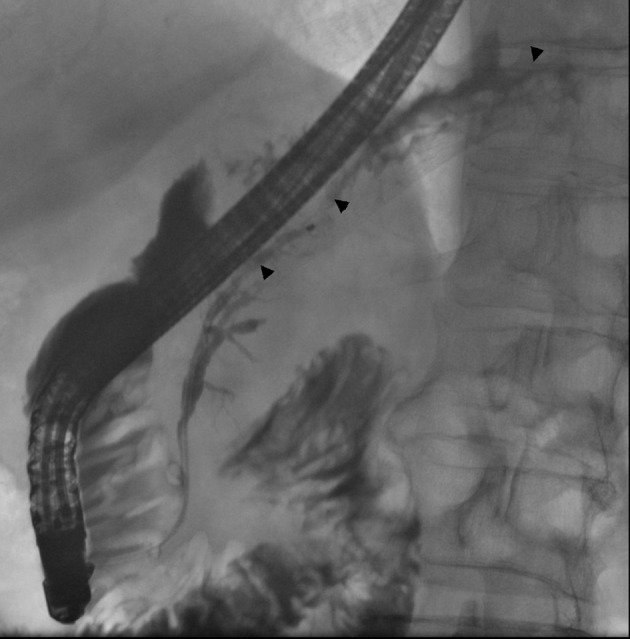
In ERCP control images, a normal pancreatic duct is seen (arrowheads) as there is no interruption in the body-tail junction and the duct of the tail fills normally. There is no sign of obvious contrast leakage. The stent has been removed.

**Figure 9:**
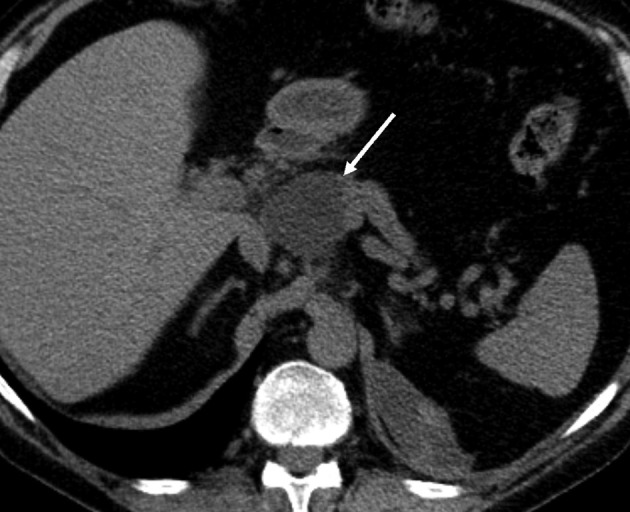
Latest CT scan image taken in the Central Hospital without contrast media shows that the pseudocyst above the pancreatic body is stable (arrow) and no new fluid collections have emerged.

The patient was admitted to the university hospital for a control CT (Fig. 7) and ERCP three months after his previous acute episodes. He was in good condition and symptom free. Laboratory tests showed a CRP of 36 mg/l and his P-amylase, alanine transaminase, alkaline phosphatase and serum bilirubin measurements were all within normal ranges. On ERCP, the pancreatic stent was removed and ductography was performed (Fig. 8). The pancreatic duct was no longer communicating with the pseudocyst and a normal pancreatic duct was seen. The wire was inserted up to the duct in the pancreas tail through the old fistula area. There was no more need for stenting. The patient went home and one control CT was scheduled at the central hospital for two months later. In that CT the pancreatic duct was normal and the fluid collection around the pancreas was the same size as that seen three months before. The left fluid collection under the diaphragm was separated and deemed to be chronic hematoma as before (Fig. 9). The patient was symptom free and no new CT scans were scheduled. Laboratory blood tests were planned for three months later.

## Discussion

Internal pancreatic fistulas are well-recognized complications of acute and chronic pancreatitis and trauma. These fistulas originate from a disruption in a major pancreatic duct, allowing the escape of exocrine secretions. If the secretions are contained in the retroperitoneum, they can burrow upwards along the diaphragmatic crura and escape into the thorax. Communications between the pancreas and the bronchial tree are rare and unusual examples of internal pancreatic fistulas [3-5]. Although the pancreatic enzymes present in the bronchial tree are not activated, they are irritating to the bronchial mucosa and promote tracheobronchitis [1].

In our patient, an internal pancreatic fistula to the tracheobronchial tree was preceded by a history of alcohol abuse but without documented inflammatory disease of the pancreas. The pancreatic fistula also followed blunt injury to left upper abdomen with a traumatic hemothorax. Trauma leading to disruption of the pancreatic duct can also cause an internal pancreatic fistula and is the most common cause in children [6]. When adherence of the pleura of the lung to the diaphragm is present, a pancreaticobronchial fistula and lung abscess would result [3].

In our case it is unclear how the previous trauma was associated with pancreatic pseudocyst formation. Perhaps there was a small disruption in the pancreatic duct and pseudocyst formation afterwards. Later, a leak from an incompletely formed or ruptured pseudocyst passed through the esophageal hiatus into the mediastinum to form a pleural fistula.

The presenting symptoms of pancreaticobronchial fistula direct attention away from the abdomen and toward the respiratory tree. Cough, dyspnea, and copious sputum production usually accompanies the appearance of the bronchial fistula [4]. Our patient was first treated by pneumathologist because of dyspnea, cough and fever. He also had copious, frothy sputum caused by the direct flow of pancreatic juice through the fistula. Determination of amylase activity in secretions was not done because the patient did not have pancreatitis and a pancreaticobronchial fistula is such a rare diagnosis.

The diagnosis of a pancreaticopleural fistula generally relies on imaging. It is suggested by the findings of several imaging modalities such as sonogram, ERCP, CT, MRCP and bronchoscopy, despite which the reported detection rate is not high. A CT scan is often the initial pancreatic imaging study obtained in these patients. Abdominal film may show calcification of the pancreas suggestive of chronic pancreatitis and can aid the diagnosis of PPF [1, 7]. In our case, CT confirmed the diagnosis of pancreaticobronchial fistula, which was maybe related to the timing of the imaging. The CT was done five months after MRCP. Although MRCP has been shown to be useful for detecting PPF [7, 8], it did not show the pancreaticobronchial fistula in our patient. It did show the pancreatic ductal disruption and large cysts but not the fistula.

ERCP is critical in confirming the diagnosis of PPF. However, the reported accuracy of ERCP in diagnosing fistulas is highly variable, ranging from 0% to 100%, with the variability likely related to the timing of the procedure and anatomic variations such as downstream strictures, pancreas divisum, and the success in cannulation of the pancreatic duct [7, 9-11].

In our patient, the first ERCP showed the ductal disruption but it was not until a new ERCP was done after five months that a fistula communicating with a mediastinal pseudocyst was seen. Treatment consisted of placement of a pancreatic stent, which facilitated internal drainage of pancreatic fluid, thus promoting healing of the fistula.

Varadarajulu et al [12] showed with multivariate analysis that 56% of pancreatic leaks resolved with pancreatic stenting and showed that a partially disrupted duct and stent bridging the disruption were associated with a successful outcome for pancreatic stenting. No recurrence during a 2-year follow-up period was noticed in study that showed that 58% of pancreatic leaks resolved with stenting [13]. Complete disruption of the pancreas duct is less amenable to endoscopic therapy [10]. Tauseef et al [7] concluded that patients with single strictures were managed successfully with pancreatic stent placement, whereas patients of multiple strictures, complete duct disruption, or large cysts were managed with surgery. The most common surgical procedure reported was distal pancreatectomy followed by pancreaticojejunostomy.

In our patient the pancreatic duct was partially disrupted. This was confirmed in the last ERCP when the stent was removed after 6 months of treatment. By ductography, the fistula had disappeared and the old leak area was crossed with a wire up to the duct in the tail of the pancreas. In the final CT made three months later, the pancreatic duct looked normal and more importantly the patient was finally symptom free.

Respiratory symptoms such as coughing, sputum, dyspnea and mild fever are typical signs of a pancreaticobronchial fistula indicating linkage from the pancreatic pseudocyst system below and above the diaphragm to the bronchial tree. It is very rare that old trauma can cause pancreatic duct leakage and later pseudocyst formation and leaks into the mediastinum. When a patient with pancreatic pseudocysts has no chronic or acute pancreatitis, it is justified to determine the medical history thoroughly. Some the reason for caught, dyspnea and fever can also be in abdomen.
